# Pregnancy-related factors and the risk of breast carcinoma *in situ *and invasive breast cancer among postmenopausal women in the California Teachers Study cohort

**DOI:** 10.1186/bcr2589

**Published:** 2010-06-18

**Authors:** Huiyan Ma, Katherine D Henderson, Jane Sullivan-Halley, Lei Duan, Sarah F Marshall, Giske Ursin, Pamela L Horn-Ross, Joan Largent, Dennis M Deapen, James V Lacey, Leslie Bernstein

**Affiliations:** 1 Department of Population Sciences, Beckman Research Institute, City of Hope, 1500 East Duarte Road, Duarte, CA 91010, USA; 2 Department of Epidemiology, School of Medicine, University of California, Irvine, 224 Irvine Hall, Irvine, CA 92697, USA; 3 Department of Preventive Medicine, Keck School of Medicine/Norris Cancer Center, University of Southern California, 1441 Eastlake Avenue, Los Angeles, CA 90033, USA; 4 Department of Nutrition, University of Oslo, PO Box 1046, Blindern, 0316 Oslo, Norway; 5 Cancer Prevention Institute of California, 2201 Walnut Avenue, Suite 300, Fremont, CA 94538, USA

## Abstract

**Introduction:**

Although pregnancy-related factors such as nulliparity and late age at first full-term pregnancy are well-established risk factors for invasive breast cancer, the roles of these factors in the natural history of breast cancer development remain unclear.

**Methods:**

Among 52,464 postmenopausal women participating in the California Teachers Study (CTS), 624 were diagnosed with breast carcinoma *in situ *(CIS) and 2,828 with invasive breast cancer between 1995 and 2007. Multivariable Cox proportional hazards regression methods were used to estimate relative risks associated with parity, age at first full-term pregnancy, breastfeeding, nausea or vomiting during pregnancy, and preeclampsia.

**Results:**

Compared with never-pregnant women, an increasing number of full-term pregnancies was associated with greater risk reduction for both breast CIS and invasive breast cancer (both *P *trend < 0.01). Women having four or more full-term pregnancies had a 31% lower breast CIS risk (RR = 0.69, 95% CI = 0.51 to 0.93) and 18% lower invasive breast cancer risk (RR = 0.82, 95% CI = 0.72 to 0.94). Parous women whose first full-term pregnancy occurred at age 35 years or later had a 118% greater risk for breast CIS (RR = 2.18, 95% CI = 1.36 to 3.49) and 27% greater risk for invasive breast cancer (RR = 1.27, 95% CI = 0.99 to 1.65) than those whose first full-term pregnancy occurred before age 21 years. Furthermore, parity was negatively associated with the risk of estrogen receptor-positive (ER+) or ER+/progesterone receptor-positive (PR+) while age at first full-term pregnancy was positively associated with the risk of ER+ or ER+/PR+ invasive breast cancer. Neither of these factors was statistically significantly associated with the risk of ER-negative (ER-) or ER-/PR- invasive breast cancer, tests for heterogeneity between subtypes did not reach statistical significance. No clear associations were detected for other pregnancy-related factors.

**Conclusions:**

These results provide some epidemiologic evidence that parity and age at first full-term pregnancy are involved in the development of breast cancer among postmenopausal women. The role of these factors in risk of *in situ *versus invasive, and hormone receptor-positive versus -negative breast cancer merits further exploration.

## Introduction

Although pregnancy-related factors such as nulliparity and late age at first full-term pregnancy are well-established risk factors for invasive breast cancer, it remains unclear whether such factors have similar effects on breast carcinoma *in situ *(CIS) or whether their effects vary across the subtypes of invasive breast cancer defined by the estrogen receptor (ER) or the joint ER and progesterone receptor (PR) status of the tumor. The clarification of these issues may shed light on a deeper understanding of the roles of pregnancy-related factors in the natural history of breast cancer development.

Breast CIS includes ductal carcinoma *in situ *(DCIS), lobular carcinoma *in situ *(LCIS), and other relatively rare forms of breast CIS, with DCIS being the predominant subtype. The term '*in situ' *indicates that neoplastic cells are present but have not spread past the boundaries of ducts or lobules where the tumor initially developed. Estimates of the percentage of patients with DCIS that will progress to invasive disease over a period of 10 or more years vary widely, ranging from 14 to 53% [1] and likely depend on a variety of factors, including type and extent of treatment and medical surveillance. Unlike DCIS, LCIS indicates neoplastic changes in the breast lobules, a precursor lesion that carries an elevated risk of invasive lobular carcinoma in either breast; thus, it is not included in clinical definitions of *in situ *breast cancer [2,3]. In contrast, DCIS and other forms of breast CIS are clinically considered pre-invasive lesions that can be associated with the development of invasive breast cancer at the same location in the breast where the CIS is located. Nevertheless, epidemiologic studies have shown that, compared with the general population, the risk of invasive breast cancer increases over four-fold following a diagnosis of either DCIS or LCIS [4,5].

Given that breast CIS is a potential precursor to invasive disease, one would expect that breast CIS and invasive breast cancer would share risk factors involved in the early stages of breast cancer development while factors affecting disease progression would be associated only with invasive breast cancer risk. Few studies have made direct comparisons of the consistency of risk estimates for pregnancy-related risk factors between breast CIS and invasive breast cancer within a study population, particularly among women whose reproductive years have ended. In studies that provided results for postmenopausal women, parity was associated with a decreased risk for both postmenopausal breast CIS and postmenopausal invasive breast cancer; results for age at first full-term pregnancy were inconsistent [6-8]. None of these studies provided results on the effects of breastfeeding, nausea or vomiting during pregnancy, or preeclampsia on the risk of breast CIS or invasive breast cancer.

Parity, early age at first full-term pregnancy, and breastfeeding influence the risk of breast cancer predominantly through hormonal mechanisms that involve estrogen and progesterone [9,10]. Previous studies have also linked severe nausea/persistent vomiting during pregnancy to elevated serum estradiol levels [11], and have shown that preeclampsia is associated with lower maternal serum levels of estriol [12] and insulin-like growth factors [13]. Therefore, severe nausea/persistent vomiting during pregnancy might be associated with an increased risk while preeclampsia might be associated with a decreased risk of breast cancer. Although a number of epidemiologic studies have examined severe pregnancy nausea/vomiting [14-16] or history of preeclampsia [17-23] in relation to invasive breast cancer risk, the data have been inconsistent for both factors. Further, no data have been published specifically for breast CIS.

Both a systematic review [24] and a meta-analysis [25] show that nulliparity and late age at first full-term pregnancy are associated with increased risk of ER-positive (ER+) or ER+/PR+, but not with ER-negative (ER-) or ER-/PR- invasive breast cancer. In contrast, the protection from breastfeeding does not differ by ER/PR status. However, these results are based largely on results from case-control studies and only a small number of these studies provide data for postmenopausal women separately from premenopausal women. Three prospective cohort studies that have assessed reproductive factors in relation to hormone receptor subtypes of invasive breast cancer provide consistent results for parity, but inconsistent results for late age at first full-term pregnancy. Further, no data are given for the association of breastfeeding with the hormone receptor subtypes of invasive breast cancer [26-28].

Thus, important questions remain regarding the roles of pregnancy-related factors in the development of breast CIS and invasive breast cancer among women who have completed their reproductive years. The authors examined the associations of parity, age at first full-term pregnancy, duration of breastfeeding, nausea or vomiting during pregnancy, and preeclampsia with breast CIS and invasive breast cancer (overall and by hormonal receptor subtype) among postmenopausal women participating in the prospective California Teachers Study (CTS) cohort.

## Materials and methods

### Study population and data collection

Details of the CTS have been described previously [29]. Briefly, the CTS is a prospective study of 133,479 current and retired female California public school teachers and administrators who were recruited from active members of the California State Teachers Retirement System. New diagnoses of first primary breast CIS or invasive breast cancer among cohort members were identified through annual linkages from 1995 through 2007 with the California Cancer Registry (CCR), a legally mandated statewide population-based cancer reporting system. Modeled after the National Cancer Institute's Surveillance, Epidemiology, and End Results (SEER) Program, the CCR is composed of three SEER Program registries and maintains the highest standards for data quality and completeness. CCR ascertainment of newly diagnosed cancers is estimated to be 99% complete [30].

All information on pregnancy-related factors except nausea or vomiting during pregnancy and preeclampsia was collected in the questionnaire completed by participants when they joined the cohort. Participants reported information on race/ethnicity, family history of breast cancer, age at menarche, detailed pregnancy (including ages at and outcomes for each pregnancy) and breastfeeding histories, menopausal status, use of menopausal hormone therapy, height, and weight. Participants reported information regarding nausea or vomiting during pregnancy and preeclampsia in the second questionnaire completed in 1997-1998. Preeclampsia was defined as a condition that can occur during the second half of a pregnancy and is marked by elevated blood pressure, protein in the urine and fluid retention.

For the current analysis, using information provided when the cohort was formed, 8,867 women who were not California residents were excluded, followed by 6,350 women who had a prior history of breast CIS or invasive breast cancer or an unknown cancer history and by 5,107 women who were 80 years or older. This yielded a preliminary analytic cohort of 113,155 women. In order to ensure that each woman's complete pregnancy history was covered, eligibility for this analysis was restricted to women who were postmenopausal at cohort entry (n = 53,440). Menopausal status was determined after reviewing a woman's age at last menstrual period, reason for cessation of menstrual periods, hysterectomy status, oophorectomy status and current use of hormonal therapy. Women were considered to be postmenopausal if they reported a natural menopause more than six months before completing the baseline questionnaire (n = 31,207), if they had a bilateral oophorectomy (n = 10,868), if they were 56 years or older and not menstruating regularly (n = 6,675), or if their periods had stopped due to other reasons including pituitary adenoma, medication, chemotherapy, radiation treatment, or another reason (n = 4,690). The age criterion was based on previous work indicating that among those with natural menopause 97% were postmenopausal by age 56 years [31]. Further exclusions were women who were missing information on age at menarche (n = 217), pregnancy history (n = 75), or both factors (n = 684). Therefore, 52,464 postmenopausal women remained in the analytic cohort.

Person-time of follow-up for each woman began with the date she completed the baseline questionnaire and ended with the first of the following events: a breast cancer diagnosis (n = 624 breast CIS including 51 women diagnosed with LCIS, ICD-O morphology code = 8520; n = 2,828 invasive), a permanent move outside of California (n = 4,481), death (n = 5,852), or December 31, 2007 (n = 38,679).

The analytic cohort included 8,937 never-pregnant and 43,527 ever-pregnant women. Of the 43,527 ever-pregnant women, 371 experienced pregnancy but did not provide outcome, and 1,957 had only incomplete pregnancies. Women who experienced pregnancy and did not provide an outcome and those who had only incomplete pregnancies were included when testing the effect of any pregnancy (ever vs. never), but were excluded from analyses of parous women. The effect of incomplete pregnancy on breast cancer risk has previously been studied in this cohort [32].

The 41,199 women who had at least one stillbirth or live childbirth were referred to as parous throughout this article. Still and live births were combined to obtain the total number of full-term pregnancies. Parous women who were missing information on breastfeeding or age at first full-term pregnancy (n = 117) were additionally excluded from analyses restricted to parous women. Analyses of parous women were based on 41,082 women of whom 479 were diagnosed with breast CIS and 2,193 were diagnosed with invasive breast cancer during follow-up.

Of 43,527 ever-pregnant postmenopausal women at cohort entry, 32,084 (73.7%; including 394 with breast CIS and 1,794 with invasive breast cancer) provided information on nausea or vomiting during any pregnancy and 31,459 (72.3%; including 391 with breast CIS and 1,768 with invasive breast cancer) provided information on preeclampsia in the CTS' second questionnaire, which was mailed in 1997, and were included in the analysis of these factors.

Since 1990, National Cancer Institute designated SEER Program registries, including those comprising the CCR, routinely abstract ER and PR status from the medical records following a breast cancer diagnosis. Among 2,828 invasive breast cancers diagnosed in the current analytic cohort, ER status was available for 2,443 (86.4%; 2,104 ER+, 339 ER-). Both ER status and PR status were available for 2,326 (82.2%; 1,651 ER+/PR+, 317 ER-/PR-, 340 ER+/PR-, 18 ER-/PR+).

The use of CTS participants' data for this analysis was approved by the Institutional Review Boards at the City of Hope, the University of Southern California, the Cancer Prevention Institute of California (formerly the Northern California Cancer Center), and the University of California at Irvine, and by the Committee for the Protection of Human Subjects, California Health and Human Services Agency.

### Data analyses

The relative hazard (represented as the relative risk (RR) and its 95% confidence interval (CI)) for the association between pregnancy-related factors and breast cancer risk was estimated using multivariable Cox proportional hazards regression methods [33]. Separate analyses were conducted for breast CIS and invasive breast cancer (the latter, overall and by hormone receptor subtypes: ER+ and ER-, ER+/PR+ and ER-/PR-).

For all analyses of breast CIS, women diagnosed with invasive breast cancer during follow-up were excluded as the diagnosis of invasive disease presumes they have passed through the *in situ *disease stage undetected. For analyses of invasive breast cancer, women who developed breast CIS were censored on the date of this diagnosis. For analyses of each receptor subtype of invasive breast cancer, women who developed all other receptor subtypes or had an unknown subtype were censored on the date of this diagnosis.

In the Cox regression models, the time scale was defined by age (in days) at cohort entry and age (in days) at event or censoring (exit). All multivariable models were adjusted for the following factors, selected *a priori*, as potential confounders: race (white, African-American, others), family history of breast cancer in a first degree relative, that is, mother, father, sister or brother (yes, no, unknown/adopted), age at menarche (< 13, ≥ 13 years), menopausal hormone therapy (HT) use (never use, ever use: only estrogen therapy (ET), only estrogen in combination with progesterone therapy (E+P), both types of HT, unknown type of HT) and body mass index (< 25, 25 to 29.9, ≥ 30 kg/m^2^, unknown).

Multivariable models for parous women were additionally mutually adjusted for number of full-term pregnancies (1, 2, 3, ≥ 4), age at first full-term pregnancy (< 21, 22 to 24, 25 to 29, 30 to34, ≥ 35 years), and duration of breastfeeding (never, < 6, 6 to 11, 12 to 23, ≥ 24 months).

The associations of breast CIS or invasive breast cancer with nausea or vomiting during pregnancy or preeclampsia were explored among postmenopausal women with at least one pregnancy as reported at cohort entry who had complete information on one or both of these variables in the second mailed questionnaire. Nausea or vomiting during any pregnancy was examined as was the number of pregnancies during which the ever-pregnant woman experienced these symptoms (1, 2, 3, ≥ 4) and whether treatment was received for these symptoms during the most recent pregnancy or during other pregnancies. Preeclampsia was evaluated as never occurring during a pregnancy, occurring during the most recent pregnancy, or not occurring during the most recent pregnancy but having occurred during at least one of other pregnancies. Multivariable models for nausea or vomiting during pregnancy or preeclampsia were additionally adjusted for pregnancy history (number of full-term pregnancies: 1, 2, 3, ≥ 4; ever pregnant but outcomes unknown; ever pregnant but no full-term pregnancy).

Tests for trend were conducted to examine the dose-response relationship of breast CIS or invasive breast cancer with number full-term pregnancies, age at first full-term pregnancy, duration of breastfeeding, and number of pregnancies during which participant experienced nausea or vomiting by fitting ordinal values corresponding to each exposure category in the statistical models and determining whether the slope parameter differed from zero using the Wald test [34].

Tests for homogeneity (evaluating the null hypothesis of homogeneity) of RR estimates were performed (for dichotomous variables or ordinal variables) by constructing a Z test of the differences in log RR divided by the square root of the sum of the variances of the two log RR estimates for the following comparisons: breast CIS vs. invasive breast cancer, ER+ vs. ER-, and ER+/PR+ vs. ER-/PR- breast cancer.

The analyses for breast CIS were repeated among 44,671 (90%) postmenopausal women who reported having their most recent mammogram less than three years before the CTS baseline survey. The results remained the same. The analysis for breast CIS was also repeated after excluding 51 women diagnosed with LCIS. Again, the results remained essentially identical. The results for breast CIS including LCIS from all postmenopausal women without invasive breast cancer (n = 49,636) are presented.

Two-sided *P*-values are reported for tests for trend and for homogeneity of RR estimates. All statistical analyses were performed using SAS version 9.1 (SAS Institute, Cary, North Carolina, USA).

## Results

### Characteristics

Women in the analytic cohort were followed for an average of 10.5 years. Table 1 provides the age-adjusted distribution of several participant characteristics according to pregnancy history (never-pregnant, ever-pregnant), age at first full-term pregnancy, and number of full-term pregnancies. Compared with never-pregnant women, those who had ever been pregnant were more likely to be African-American women. Parous African-American women were less likely than other women to have had three or more full-term pregnancies with the first full-term pregnancy at age 25 years or older. Parous women who had more full-term pregnancies (≥ 3) were more likely to have a longer duration of breastfeeding than those who had fewer full-term pregnancies (< 3).

**Table 1:** Age-adjusted^a ^percent distribution of baseline characteristics

	Total	Ever pregnant, %		Parous women (number of full-term pregnancy/age at first full-term pregnancy, yrs), %			
			
		No	Yes	< 3/< 25	< 3/≥ 25	≥ 3/< 25	≥ 3/≥ 25
		(n = 8,937)	(n = 43,527)	(n = 8,034)	(n = 14,825)	(n = 10,851)	(n = 7,372)
Race							
White	46,624	16.8	83.2	19.8	34.4	28.2	17.7
African-American	1,622	11.1	88.9	25.9	34.6	28.3	11.2
Others	4,218	17.2	82.8	17.7	36.0	27.9	18.4
							
First-degree family history of breast cancer							
No	43,489	16.6	83.4	19.8	34.5	28.3	17.3
Yes	7,132	16.8	83.2	19.2	34.5	27.0	19.3
Unknown	1,843	17.8	82.2	20.8	35.7	27.7	15.9
							
Age at menarche, years							
< 13	26,220	16.9	83.1	20.6	34.3	28.7	16.4
≥ 13	26,244	16.4	83.6	19.0	34.8	27.6	18.7
							
BMI, kg/m^2^							
< 25	27,583	16.9	83.1	20.4	35.8	26.6	17.2
25 to 29.9	14,354	15.8	84.2	19.1	32.6	30.0	18.3
≥ 30	7,783	17.5	82.5	19.1	33.4	30.6	16.9
Unknown	2,744	16.0	84.0	18.7	35.6	26.7	19.0
							
Menopausal HT use							
Never	10,945	20.4	79.6	16.0	35.5	27.3	21.2
E only	15,676	15.6	84.4	21.8	31.2	30.4	16.7
E plus P only	14,959	15.8	84.2	19.8	37.7	25.8	16.7
Mixed use	7,169	15.7	84.3	21.1	32.7	30.3	15.9
Unknown type of HT	3,715	15.2	84.8	19.8	35.7	26.7	17.8
							
Duration of breastfeeding for parous women, months							
Never	12,628	-	-	25.0	39.7	22.0	13.3
< 6	11,202			24.1	35.2	26.8	13.9
6 to 11	7,124			19.5	38.2	26.9	15.4
12 to 23	6,486			11.1	28.4	35.3	25.2
≥ 24	3,642			3.3	17.2	44.2	35.3

Results are for 52,464 postmenopausal women in the California Teachers Study followed between 1995 and 2007.^a^Age distribution in each category of the baseline characteristic variables was adjusted according to age distribution of the 52,464 participants. Abbreviations: BMI, body mass index; HT, hormone therapy; E, estrogen; P, progestin.

### Parity, age at first full-term pregnancy and duration of breastfeeding

Compared with never-pregnant women, women who experienced any pregnancy had reduced risks of breast CIS (RR = 0.85, 95% CI = 0.69 to 1.04) and invasive breast cancer (RR = 0.91, 95% CI = 0.83 to 1.00) (Table 2). Compared with never-pregnant women, the magnitude of risk reductions increased with increasing number of full-term pregnancies for both breast CIS (*P *trend = 0.008) and invasive breast cancer (*P *trend = 0.003).

**Table 2:** Adjusted RRs for the association between pregnancy-related factors and breast CIS and invasive breast cancer

	Breast CIS			Invasive breast cancer		
		
	Observed Person-years	Cases No.	Adjusted RR (95% CI)	Observed Person-years	Cases No.	Adjusted RR (95% CI)
**All women**						
Ever pregnant						
No	90,296	116	Reference	92,927	493	Reference
Yes	444,276	508	0.85 (0.69 to 1.04)	457,417	2335	0.91 (0.83 to 1.00)
*P *for homogeneity^a^			*0.55*			
						
Number of full-term pregnancies^b^						
1	68,527	91	1.01 (0.77 to 1.33)	70,615	355	0.95 (0.83 to 1.09)
2	165,472	188	0.84 (0.67 to 1.06)	170,385	878	0.94 (0.84 to 1.04)
3	112,458	131	0.84 (0.65 to 1.08)	115,629	591	0.88 (0.78 to 0.99)
≥ 4	74,458	70	0.69 (0.51 to 0.93)	76,634	373	0.82 (0.72 to 0.94)
*P trend*			*0.008*			*0.003*
*P *for homogeneity^a ^			*0.26*			
Ever pregnant, incomplete pregnancies only or outcome of pregnancies unknown	23,361	28		24,154	138	
						
**Parous women only**^c, d^						
Number of full-term pregnancies						
1	68,338	90	Reference	70,426	355	Reference
2	164,995	188	0.92 (0.70 to 1.20)	169,889	875	1.00 (0.88 to 1.14)
3	112,082	131	0.93 (0.69 to 1.26)	115,253	590	0.95 (0.82 to 1.10)
≥ 4	74,291	70	0.77 (0.54 to 1.11)	76,467	373	0.90 (0.77 to 1.07)
*P trend*			*0.22*			*0.15*
*P *for homogeneity^a ^			*0.61*			
						
Age at first full-term pregnancies, years						
< 21	46,965	44	Reference	48,165	203	Reference
21 to 24	146,568	166	1.17 (0.84 to 1.63)	150,692	727	1.07 (0.92 to 1.25)
25 to 29	158,816	183	1.18 (0.84 to 1.66)	163,749	888	1.22 (1.05 to 1.43)
30 to 34	51,266	50	0.99 (0.65 to 1.50)	52,784	281	1.22 (1.01 to 1.47)
≥ 35	16,091	36	2.18 (1.36 to 3.49)	16,644	94	1.27 (0.99 to 1.65)
*P trend*			*0.09*			*0.002*
*P *for homogeneity^a ^			*0.82*			
						
Duration of breastfeeding, months						
Never	127,851	151	Reference	131,753	688	Reference
< 6	113,869	132	0.97 (0.77 to 1.22)	117,111	571	0.94 (0.84 to 1.05)
6-11	73,115	82	0.93 (0.71 to 1.22)	75,315	402	1.05 (0.93 to 1.19)
12-23	67,172	79	1.03 (0.77 to 1.36)	69,112	352	1.02 (0.90 to 1.17)
≥ 24	37,699	35	0.87 (0.59 to 1.29)	38,744	180	0.99 (0.84 to 1.18)
*P trend*			*0.70*			*0.56*
*P *for homogeneity^a ^			*0.56*			

RRs are from multivariable Cox proportional hazards regression models using age (in days) as the time metric and stratified by age (in years) with the adjustment for race, family history of breast cancer in a first degree relative, age at menarche, HT use, body mass index. ^a^Homogeneity in risk estimates between breast CIS and invasive breast cancer. ^b^Compared with nulligravid women. ^c^Additionally, number of full-term pregnancies, age at first full-term pregnancy, and duration of breastfeeding mutually adjusted among parous women. ^d^Additionally excluded parous women who were missing information on breastfeeding or age at first full-term pregnancy. Abbreviations: CIS, carcinoma *in situ*; RR, relative risk; CI, confidence interval; HT, hormone therapy.

In analyses restricted to parous women, increasing age at first full-term pregnancy was associated with an increased risk for both breast CIS and invasive breast cancer (breast CIS, *P *trend = 0.09; invasive breast cancer, *P *trend = 0.002) (Table 2). Women who had a first full-term pregnancy at age 35 years or older had more than a two-fold greater risk of breast CIS (RR = 2.18, 95% CI = 1.36 to 3.49), and 27% (RR = 1.27, CI = 0.99 to 1.65) greater risk of invasive breast cancer compared with women whose first full-term pregnancy was before age 21 years. In a model adjusted for age at first full-term pregnancy and number of full-term pregnancies, duration of breastfeeding was not statistically significantly associated with the risk of either breast CIS or invasive breast cancer among parous women.

Further, the risk estimates for parity, age at first full-term pregnancy, and breastfeeding did not differ statistically between breast CIS and invasive breast cancer (all *P *for homogeneity > 0.25). In addition, these risk estimates for both breast CIS and invasive breast cancer did not vary within subgroups defined by body mass index (< 25, ≥ 25 kg/m^2^), HT use (never, ever use), and race (white, others).

In the analyses for invasive breast cancer by ER status and joint ER and PR status, ever being pregnant, having multiple full-term pregnancies, and having an early age at first full-term pregnancy were each statistically significantly associated with reduced risk of ER + and of ER +/PR + invasive breast cancer, and were not statistically significantly associated with ER- or ER-/PR- invasive breast cancer, but none of the tests for homogeneity of risk estimates comparing results for ER+ to those for ER- or comparing those for ER +/PR + to those for ER -/PR - reached statistical significance (Table 3). Duration of breastfeeding was not statistically significantly associated with anyone of ER +, ER -, ER+/PR +, and ER -/PR - invasive breast cancer.

**Table 3:** Adjusted RRs for the association between pregnancy-related factors and the subtypes of invasive breast cancer

	ER+		ER-		ER+/PR+		ER-/PR-	
				
	Cases No.	Adjusted RR (95% CI)	Cases No.	Adjusted RR (95% CI)	Cases No.	Adjusted RR (95% CI)	Cases No.	Adjusted RR (95% CI)
**All women**								
Ever pregnant								
No	382	Reference	56	Reference	318	Reference	50	Reference
Yes	1,722	0.87 (0.78 to 0.97)	283	0.99 (0.74 to 1.31)	1,333	0.81 (0.71 to 0.91)	267	1.04 (0.77 to 1.41)
*P *for homogeneity^a^		*0.42*				*0.12*		
								
Number of full-term pregnancy^b^								
1	272	0.93 (0.80 to 1.09)	33	0.78 (0.51 to 1.20)	218	0.90 (0.75 to 1.06)	32	0.85 (0.55 to 1.33)
2	640	0.88 (0.77 to 1.00)	121	1.15 (0.84 to 1.58)	485	0.80 (0.69 to 0.92)	111	1.19 (0.85 to 1.66)
3	443	0.85 (0.74 to 0.98)	66	0.88 (0.62 to 1.26)	352	0.82 (0.70 to 0.95)	66	0.99 (0.68 to 1.43)
≥ 4	267	0.76 (0.65 to 0.89)	45	0.90 (0.61 to 1.34)	206	0.71 (0.59 to 0.85)	40	0.90 (0.59 to 1.37)
*P trend*		*0.0004*		*0.72*		*< 0.0001*		*0.85*
*P *for homogeneity^a^		*0.33*				*0.17*		
Ever pregnant, incomplete pregnancies only or outcome of pregnancies unknown	100		18		72		18	
								
**Parous women only**^c, d^								
Number of full-term pregnancies								
1	272	Reference	33	Reference	218	Reference	32	Reference
2	638	0.96 (0.83 to 1.11)	121	1.51 (1.01 to 2.25)	484	0.91 (0.77 to 1.08)	111	1.39 (0.92 to 2.10)
3	443	0.95 (0.80 to 1.12)	65	1.18 (0.75 to 1.86)	352	0.95 (0.79 to 1.15)	65	1.17 (0.74 to 1.85)
≥ 4	267	0.85 (0.70 to 1.04)	45	1.24 (0.75 to 2.07)	206	0.84 (0.68 to 1.05)	40	1.08 (0.64 to 1.83)
*P trend*		*0.13*		*0.99*		*0.25*		*0.74*
*P *for homogeneity^a^		*0.57*				*0.87*		
								
Age at first full-term pregnancy, years								
< 21	152	Reference	22	Reference	120	Reference	21	Reference
21 to 24	527	1.04 (0.87 to 1.25)	97	1.37 (0.86 to 2.18)	413	1.04 (0.85 to 1.28)	90	1.31 (0.81 to 2.12)
25 to 29	660	1.21 (1.01 to 1.45)	98	1.31 (0.82 to 2.10)	501	1.18 (0.97 to 1.45)	93	1.28 (0.79 to 2.07)
30 to 34	204	1.17 (0.94 to 1.46)	39	1.71 (0.99 to 2.95)	166	1.23 (0.96 to 1.57)	37	1.66 (0.95 to 2.90)
≥ 35	77	1.37 (1.02 to 1.83)	8	1.21 (0.52 to 2.81)	60	1.36 (0.98 to 1.89)	7	1.07 (0.44 to 2.60)
*P trend*		*0.004*		*0.23*		*0.009*		*0.30*
*P *for homogeneity^a^		*0.96*				*0.92*		
								
Duration of breastfeeding, months								
Never	499	Reference	80	Reference	379	Reference	75	Reference
< 6	433	0.98 (0.86 to 1.11)	68	0.97 (0.70 to 1.34)	346	1.03 (0.89 to 1.19)	63	0.96 (0.69 to 1.34)
6 to 11	296	1.06 (0.92 to 1.23)	52	1.15 (0.81 to 1.63)	229	1.08 (0.91 to 1.27)	50	1.16 (0.81 to 1.67)
12 to 23	259	1.04 (0.89 to 1.21)	42	1.02 (0.69 to 1.50)	205	1.08 (0.90 to 1.29)	39	1.02 (0.69 to 1.52)
≥ 24	133	1.02 (0.83 to 1.25)	22	1.00 (0.61 to 1.66)	101	1.00 (0.80 to 1.27)	21	1.06 (0.64 to 1.78)
*P trend*		*0.52*		*0.78*		*0.54*		*0.65*
*P *for homogeneity^a^		*0.98*				*0.88*		

RRs are from multivariable Cox proportional hazards regression models using age (in days) as the time metric and stratified by age (in years) with the adjustment for race, family history of breast cancer in a first degree relative, age at menarche, HT use, body mass index. ^a^Homogeneity in risk estimates between subtypes of invasive breast cancer. ^b^Compared with nulligravid women. ^c^Additionally, number of full-term pregnancies, age at first full-term pregnancy, and duration of breastfeeding mutually adjusted among parous women. ^d^Additionally excluded parous women who were missing information on breastfeeding or age at first full-term pregnancy. Abbreviations: RR, relative risk; CI, confidence interval; ER, estrogen receptor; PR, progesterone receptor; HT, hormone therapy.

### Nausea or vomiting during pregnancy

Among women who experienced any pregnancy, nausea or vomiting during pregnancy was not associated with risk of breast CIS or invasive breast cancer regardless of the number of pregnancies in which it occurred (one or more) or if the woman received treatment for nausea or vomiting of pregnancy (a measure of severity of the condition) or the timing of that treatment (for the most recent or only for earlier pregnancies) (all 95% CIs include 1, all *P *trend > 0.05; Table 4).

**Table 4:** Adjusted RRs for the association between pregnancy-related conditions and breast CIS and invasive breast cancer

	Breast CIS			Invasive breast cancer			
		
	Observed Person-years	Cases No.	Adjusted RR (95% CI)	Observed Person-years	Cases No.	Adjusted RR (95% CI)	***P *for homogeneity**^**b**^
**Nausea or vomiting during pregnancy**							
Ever experienced							
No	108,269	124	Reference	111,618	620	Reference	
Yes	224,001	270	1.05 (0.85 to 1.30)	230,499	1,174	0.92 (0.84 to 1.02)	*0.29*
Number of pregnancies during which participant experienced nausea or vomiting^a^							
1	73,892	84	0.92 (0.70 to 1.22)	75,959	380	0.91 (0.80 to 1.03)	
2	72,819	95	1.21 (0.91 to 1.61)	75,015	387	0.94 (0.82 to 1.07	
3	43,520	53	0.99 (0.70 to 1.41)	44,783	238	0.94 (0.80 to 1.11)	
≥ 4	33,770	38	1.16 (0.76 to 1.77)	34,742	169	0.91 (0.75 to 1.11)	
*P trend*			*0.37*			*0.25*	*0.20*
Ever needed treatment for nausea or vomiting during pregnancy^a^							
No	184,207	220	1.04 (0.83 to 1.30)	189,367	947	0.90 (0.81 to 1.00)	*0.26*
Yes, for most recent pregnancy	19,436	24	1.09 (0.70 to 1.69)	20,092	114	1.07 (0.88 to 1.31)	*0.96*
Yes, for other pregnancy(ies)	20,358	26	1.14 (0.74 to 1.74)	21,039	113	1.01 (0.83 to 1.24)	*0.63*
							
**Preeclampsia**							
Ever diagnosed with preeclampsia							
No	304,969	361	Reference	314,176	1673	Reference	
Yes, during most recent pregnancy	8,431	17	1.72 (1.05 to 2.81)	8,699	44	0.98 (0.73 to 1.33)	*0.06*
Yes, during other pregnancy(ies)	12,379	13	0.93 (0.53 to 1.62)	12,648	51	0.77 (0.58 to 1.02)	*0.57*

RRs for gravid postmenopausal women based on multivariable Cox proportional hazards regression models using age (in days) as the time metric and stratified by age (in years) with the adjustment for race, family history of breast cancer in a first degree relative, age at menarche, HT use, body mass index, pregnancy history (number full-term pregnancies: 1; 2; 3; ≥ 4; ever pregnant, but unknown whether full-term; ever been pregnant, but not full-term pregnancy). ^a^Compared with gravid postmenopausal women who had never experienced nausea or vomiting during pregnancy. bHomogeneity in risk estimates between breast CIS and invasive breast cancer. Abbreviations: CIS, carcinoma *in situ*; RR, relative risk; CI, confidence interval; HT, hormone therapy

### Preeclampsia

Among women who experienced any pregnancy, preeclampsia diagnosed during the most recent pregnancy was associated with an increased risk of breast CIS (RR = 1.72, 95% CI = 1.05 to 2.81), but was not associated with invasive breast cancer (Homogeneity test *P *= 0.06, Table 4). Preeclampsia diagnosed during any pregnancies prior to the most recent was not statistically significantly associated with either breast CIS risk (RR = 0.93, 95% CI = 0.53 to 1.62) or invasive breast cancer (RR = 0.77, 95% RR = 0.58 to 1.02).

## Discussion

In the CTS, nulliparity and late age at first full-term pregnancy were associated with higher risk for both breast CIS and invasive breast cancer, although not all of the associations with breast CIS reached statistical significance, in part due to the lower incidence of breast CIS in the cohort. Three previous studies have reported data on both breast CIS and invasive breast cancer for women beyond reproductive age [6-8]. In a cohort study of 32,607 postmenopausal women followed for approximately five years, nulliparous women had a 70% higher risk of breast CIS and a 40% higher risk of invasive breast cancer than women who had a full-term pregnancy before age 21 years; the same study showed that later age at first full-term pregnancy was associated with an increased risk of invasive breast cancer, but was not statistically significantly associated with breast CIS risk [8]. The Iowa Women's Health Study whose 37,105 postmenopausal participants were followed for 11 years found that women with late age at first birth (≥ 30 years) had a 92% and a 29% greater risk for DCIS and invasive breast cancer, respectively, than women whose first birth occurred at or before age 20 years [6]. One previous population-based case-control study also reported that parous postmenopausal women had decreased risk of both breast CIS and invasive breast cancer relative to nulliparous women while late age at first full-term pregnancy was not associated with either breast CIS or invasive breast cancer [7]. Although the associations of parity and age at first full-term pregnancy with risk of breast CIS and invasive breast cancer have not been entirely consistent across epidemiologic studies, as a whole, the data provide some evidence that these two factors are involved in the early stages of breast cancer development.

The mechanisms underlying the protective effect of early parity on breast cancer are not yet clear. Mouse models have demonstrated that breast carcinogenesis is significantly inhibited when rats have completed one pregnancy prior to exposure to the carcinogen compared with age-matched virgin rats [35,36]. This protective effect may be attributed to permanent structural and functional changes induced in the mammary parenchyma by the reproductive process, including exposure to pregnancy hormones, resulting in a lower susceptibility of epithelial cells to future carcinogenic stimuli [37,38]. Moreover, rat gene expression data suggest that persistent pregnancy-induced changes in mammary gene expression may account for the protection of parity [39]. Human data suggest that hormonal mechanisms are involved in pregnancy-related protection against breast cancer including lowered circulating estrogen and progesterone [40-42], higher levels of sex hormone-binding globulin [40] and possibly, human chorionic gonadotropin [43], which has also been demonstrated in a rat carcinogenesis model [44].

In the CTS analyses for invasive breast cancer by hormone receptor subtype, nulliparity and late age at first full-term pregnancy were associated with an increased risk of ER + or ER +/PR + invasive breast cancer but not with ER - or ER -/PR - invasive cancer. These are essentially consistent with the conclusions of a systematic review [24] and a meta-analysis [25], both consisting largely of case-control studies. Compared with data from previous cohort studies, the CTS results for both parity and age at first full-term pregnancy are basically consistent with those reported for the Iowa Women's Health Study [28] and Women's Health Initiative Cohort [26], but the CTS results for age at first full-term pregnancy are inconsistent with those reported for the Nurses' Health Study, in which late age at first full-term pregnancy was associated with an increased risk of ER -/PR - but not ER +/PR +, tumors [27]. Therefore, similar to the majority of previous data, these results support the hypothesis that some pregnancy-related risk factors may differentially influence risk for breast cancer subtypes classified by ER and PR status.

Breastfeeding has been proposed to protect against breast cancer through hormonal mechanisms that include postponing the resumption of ovulatory menstrual cycles after a pregnancy [45], reducing estrogen levels in the breast [46], and having fully differentiated breast tissue which is less susceptible to the hormone milieu [47]. In addition, it has been proposed that breastfeeding also has a direct mechanical effect by which carcinogenic agents are excreted from the breast ductal tissue [48]. However, among parous postmenopausal CTS participants, breastfeeding was not associated with either breast CIS or invasive breast cancer (overall or by receptor subtypes). The observed reductions in breast cancer risk in many previous studies have been stronger for or restricted to younger or premenopausal women [10,15,49-52]. However, in some studies, this reduction in risk was observed in the postmenopausal years [53-55] or was negligible in both age groups [56,57]. Two studies found that the protective effect of breastfeeding decreased with the increasing time after a pregnancy [15,58]. Among the parous women ages 35 to 49 year who participated in Women's Contraceptive and Reproductive Experiences study, the odds ratio (95% CI) of invasive breast cancer associating with at least 24-month breastfeeding was 0.38 (0.19 to 0.77) among women who had given birth within five years and 0.69 (0.51 to 0.93) among those beyond five years [58]. Among postmenopausal CTS participants, the average interval between the last pregnancy and CTS baseline survey was 32 years, and only 1% of the intervals were within 15 years. Fine stratification of the interval between the last pregnancy and CTS baseline survey still did not show any association between breastfeeding and breast cancer risk (results not shown). Therefore, the lack of an association between breastfeeding and breast cancer in the CTS analyses may be due to the restriction to postmenopausal women.

Since hyperemesis gravidarum, that is, severe nausea combined with persistent vomiting during pregnancy, has been linked to an elevated serum estradiol level [11], one might expect a positive association between these events and the risk of breast CIS or invasive breast cancer. Being *treated *served as a proxy for *severe *nausea or vomiting of pregnancy, with *not treated *serving as a proxy for mild/moderate symptoms. However, no association was observed by severity of nausea or vomiting. Consistent with these results, a population-based case-control study has also reported no association of breast cancer risk overall or by age (< 50, ≥ 50) with hyperemesis gravidarum [14]. In contrast with the results presented here, one case-control study reported that having ever been treated for nausea or vomiting during pregnancy was associated with an increased risk of breast cancer [15] while another case-control study reported that nausea or vomiting during first pregnancy was associated with a slightly lower risk of breast cancer [16]. Both of these studies were conducted among young women (< 45 years of age). It is possible that the null results from the CTS study are due to an older population of women, or that the impact of this condition or the hormonal changes related to it, dissipates with time since the pregnancy.

Preeclampsia has been associated with lower maternal serum levels of estriol [12] and insulin-like growth factors [13]. Therefore, preeclampsia might be associated with a lower breast cancer risk. However, among postmenopausal women in the CTS, preeclampsia diagnosed during a woman's most recent pregnancy was associated with an increased risk of breast CIS, while preeclampsia diagnosed during any pregnancies prior to the most recent was not associated with breast CIS risk. Preeclampsia was not associated with invasive breast cancer. Although a number of epidemiologic studies have examined history of preeclampsia in relation to invasive breast cancer risk, none have reported data specifically for breast CIS. Among cohort studies reporting results for preeclampsia and invasive breast cancer, one found a 38% (95% CI = 1.00 to 1.89) greater risk of invasive breast cancer among women who ever experience preeclampsia than among women with no such history [17], while another reported that women with preeclampsia and/or hypertension diagnosed during their first pregnancies had 19% (95% CI = 0.71 to 0.91) lower risk for breast cancer than women who had not experienced preeclampsia [18]. A third cohort study reported no association [19]. In contrast, several case-control studies have reported that preeclampsia was associated with a decreased risk of breast cancer [20-23]. Based on the CTS results and those of earlier studies, no conclusion can be drawn as to whether preeclampsia is a risk factor for breast cancer.

Strengths of the current study include its size, the large number of women who have been diagnosed with an incident breast cancer, the ability to identify and confirm cancer diagnoses through the CCR, California's high-quality statewide cancer registry, and collection of pregnancy-related information prior to the diagnosis of breast CIS or invasive breast cancer.

Several limitations of the current study must be considered. ER and PR status results were collected by the regional registries in California from pathology laboratories located throughout the state; these laboratories may vary in their application of immunohistochemical methods and the cutpoints used to assign a positive status. However, it is unlikely that any methodological differences would influence the observed associations, as a large validation study comparing registry reports of receptor status to those of a single *expert *laboratory found only small differences in risk estimates for ER+/PR+ and ER-/PR- breast cancer between the two sources [59]. Although not all women with invasive breast cancer had ER or PR status available, the numbers missing this information were relatively small (14% of cases missing ER status; 18% missing joint ER and PR status), and the percentage of women with missing information gradually decreased during follow-up, both of which are similar to the rates presented in previous studies conducted within SEER registries [60,61]. Women with invasive breast cancer who had ER status available in the CCR had pregnancy-related factors (ever pregnant, number of full-term pregnancies, age at first full-term pregnancy, and duration of breastfeeding) that were similar to those of women who were missing ER status information (all Pearson's chi-square *P *> 0.07). These pregnancy-related factors were also similar between women having invasive breast cancer who had information on both receptor status markers and those without this information, except that those with the information for both ER and PR status were slightly more likely to have never been pregnant (18.1% vs. 14.1%, Pearson's chi-square *P *= 0.03). This difference could cause an overestimate of any protective effect of pregnancies on all subtypes, but it is unlikely that this bias would be restricted to ER+/PR+ invasive breast cancer. In addition, the relative risk estimates for ER+/PR+ subtype differed minimally from those for ER+ subtype and the estimates for ER-/PR- subtype were similar to those for ER- subtype. Therefore, the missing information on ER and/or PR status is unlikely to have introduced measurable bias in this study. Since 75% of women with breast CIS had no data for ER/PR status, we were unable to examine the association between pregnancy-related factors and the risk of breast CIS by ER/PR status.

Another limitation is that the analyses for nausea or vomiting during pregnancy and preeclampsia were based on approximately 73% of ever-pregnant postmenopausal women. These women were similar to those without information on these two factors in terms of race, family history of breast cancer in a first degree relative, age at menarche, HT use, body mass index, number of full-term pregnancy, age at first full-term pregnancy, and duration of breastfeeding. Therefore, selection bias is unlikely to be an important explanation for the results for these two factors.

## Conclusions

These results provide some epidemiologic evidence that parity and age at first full-term pregnancy are involved in the development of breast cancer among postmenopausal women. This study did not provide evidence of any association of breastfeeding with postmenopausal diagnosis of breast CIS or invasive breast cancer overall or by hormone receptor subtypes. Further, nausea or vomiting during pregnancy was not associated with stage of breast cancer and no evidence was observed that preeclampsia is clearly associated with lower breast cancer risk.

## Abbreviations

BMI: body mass index; CCR: California Cancer Registry; CI: confidence interval; CIS: carcinoma *in situ*; CTS: California Teachers Study; DCIS: ductal carcinoma *in situ*; E: estrogen; ER: estrogen receptor; HT: hormonal therapy; LCIS: lobular carcinoma *in situ*; P: progestin; PR: progesterone receptor; RR: relative risk; SEER: Surveillance, Epidemiology, and End Results.

## Competing interests

The authors declare that they have no competing interests.

## Authors' contributions

LB and PLHR participated in the study design and supervised the data collection. Data management was conducted by HM, JSH, and SM. HM and SM conducted data analyses under the supervision of LB. HM drafted the manuscript. All authors participated in the revision of the manuscript and have read and approved the final version.
